# 4-Bromomethcathinone (4-BMC) Revisited: The First Reported Fatal Case and Toxicological Insights

**DOI:** 10.3390/metabo16070507

**Published:** 2026-07-21

**Authors:** Karolina Nowak, Paweł Szpot, Marcin Zawadzki, Agnieszka Chłopaś-Konowałek

**Affiliations:** 1 Department of Pharmacology, Faculty of Medicine, University of Opole, 48 Oleska Street, 45052 Opole, Poland; 2 Department of Forensic Medicine, Faculty of Medicine, Wroclaw Medical University, 4 J. Mikulicza-Radeckiego Street, 50345 Wroclaw, Poland; pawel.szpot@umw.edu.pl; 3 Department of Social Sciences and Infectious Diseases, Faculty of Medicine, Wroclaw University of Science and Technology, 27 Wybrzeze Wyspianskiego Street, 50370 Wroclaw, Poland; m.zawadzki@pwr.edu.pl; 4 Department of Forensic Medicine, Division of Molecular Techniques, Wroclaw Medical University, 52 M. Curie-Sklodowskiej Street, 50369 Wroclaw, Poland; agnieszka.chlopas-konowalek@umw.edu.pl; 5 Institute of Toxicology Research, 55/61/306 M. Curie-Sklodowskiej Street, 50369 Wroclaw, Poland

**Keywords:** 4-bromomethcathinone, fatal intoxication, synthetic cathinone, biological material, forensic toxicology, UHPLC-QqQ-MS/MS

## Abstract

**Background:** 4-bromomethcathinone (4-BMC) has been present on the illicit drug market since 2011. Recent EUDA data indicate renewed availability of 4-BMC in Europe, including substantial seizures reported in 2024. Despite numerous seizures of this compound across Europe during its fourteen-year presence, no fatal intoxication involving 4-BMC has been reported so far. This paper presents the first documented fatal intoxication involving 4-BMC reported worldwide. **Methods:** Quantitative determination of 4-BMC in post-mortem blood and urine was performed using the Ultra-High Performance Liquid Chromatography coupled with Triple Quadrupole Mass Spectrometry (UHPLC-QqQ-MS/MS) method, together with complementary toxicological analyses. **Results:** The 4-BMC concentrations were 16,601 ng/mL in blood and over 50,000 ng/mL in urine. Both matrices also contained 3-chloromethcathinone at 1.7 ng/mL in blood and 122.2 ng/mL in urine. No other drugs, medications, or new psychoactive substances were detected apart from bromomethcathinone and chloromethcathinone metabolites. **Conclusions:** This first fatal case associated with 4-BMC underscores the necessity for enhanced monitoring of this compound by both forensic and clinical toxicologists. The re-emergence of 4-BMC after more than a decade demonstrates that previously known xenobiotics may reappear and again pose a serious threat to human health.

## 1. Introduction

The market of new psychoactive substances (NPSs) is characterized by the continuous appearance of novel compounds, which are designed to both imitate the pharmacological effects of controlled drugs and evade already existing legislation. The largest and most dynamic groups of NPS, which have been monitored by European drug supervision systems over the last two decades, are synthetic cathinones (SCs) and synthetic cannabinoids [1,2,3,4]. Despite the implementation of legislative measures, early warning systems and international monitoring programmes, synthetic cathinones still remain widely available. Furthermore, due to their high abuse potential, unpredictable toxicity, and rapidly changing chemical composition, SCs continue to pose a substantial public health concern as well [1,2,4].

Synthetic cathinones, also referred to as β-ketoamphetamines, are structurally related to naturally occurring cathinone, the principal psychoactive alkaloid found in *Catha edulis* (khat). In terms of chemical structure, they are closely related to classical psychostimulants such as amphetamine, methamphetamine, cocaine, and 3,4-methylenedioxymethamphetamine (MDMA) [5,6]. Depending on the manufacturer or production batch, the amount of active ingredients, impurities or concentrations may differ. That factor significantly increases the risk of intoxication and complicates toxicological interpretation [2,6].

Synthetic cathinones owe their psychostimulant properties primarily to their interactions with monoamine transporters, including dopamine transporters (DATs), norepinephrine transporters (NETs) and serotonin transporters (SERTs) [5,6]. These substances may act as monoamine reuptake inhibitors, monoamine releasers or both, as a result providing increased extracellular concentrations of dopamine, norepinephrine and serotonin within the central nervous system [5]. In addition, some synthetic cathinones may interact with vesicular monoamine transporter 2 (VMAT2), further enhancing monoaminergic neurotransmission [5,6]. Proven by recent structure–activity relationship studies, even small modifications of the structure of the compound may have a huge impact on the transporter selectivity, potency, toxicity, and abuse potential of the substance [6]. Consequently, newly emerging synthetic cathinones frequently differ significantly from previously known compounds in terms of pharmacological profiles.

Following the international scheduling of mephedrone (4-MMC), myriad structural analogues have been introduced to the illicit drug market. The purpose of putting such substances on the market is to maintain psychostimulant effects, but to simultaneously avoid legal restrictions [7,8]. Essentially, among these compounds, para-substituted cathinones represent a particularly important subgroup. Replacement of the hydrogen atom at the para position of the phenyl ring with halogen substituents led to the development of flephedrone (4-fluoromethcathinone; 4-FMC), clephedrone (4-chloromethcathinone; 4-CMC), and brephedrone (4-bromomethcathinone; 4-BMC) [7,8]. In Supplementary Materials (Figure S1) structures of 2-, 3-, and 4-isomers of methylmethcathinone, chloromethcathinone, bromomethcathinone, and fluoromethcathinone are presented.

4-bromomethcathinone (4-BMC, brephedrone) is the *para*-brominated analogue of methcathinone and a positional isomer of 2-bromomethcathinone (2-BMC) and 3-bromomethcathinone (3-BMC) [9]. In terms of structure, it is closely related to mephedrone (4-MMC) and clephedrone (4-CMC), what suggests that this compound may display comparable stimulant properties [9].

In fact, the interest in 4-BMC has enhanced recently. According to the EUDA Initial Report, approximately one tonne of 4-BMC was seized in Europe in 2024 alone, representing approximately 97% of all 4-BMC seized between 2011 and 2024 [9]. Moreover, the substance has not only lately been identified in multiple European countries, but also has become sufficiently prevalent to trigger formal EU-level risk assessment procedures [4,9]. These observations suggest a re-emergence of brephedrone within the European NPS market and that is why they should underscore the need for increased awareness among forensic toxicologists and public health authorities.

4-BMC concerning analytical information remains confined as well. Previous studies have described its determination in samples including blood [10], urine [11], seized non-biological materials [12], and, more recently, investigations of its metabolic pathways in human hepatocytes and authentic biological specimens [13]. Berardinelli et al. reported that 4-BMC undergoes extensive biotransformation and identified several metabolites. These might be used to serve as biomarkers of intake, emphasizing the importance of comprehensive analytical approaches for documenting exposure to this substance [13]. If there is a substance that has an unknown metabolic pathway, what is common for many NSP, then the detection of its metabolites in biological samples has a potent significance for the diagnosis of use or intoxication [13]. A better cognition of 4-BMC biotransformation pathways might help the toxicologists to identify the biomarkers of synthetic cathinones intake in clinical and forensic diagnostics. It can also enable the detection of these compounds for an extended period of time, compared to the primary compound analysis only. The metabolism of 4-BMC is conducted mainly by the hepatic isoenzymes of P450 cytochrome. The main I phase of transformation reactions that are common for 3-CMC, 4-CMC and 4-BMC β-ketoreduction, *N*-demetilation and the conjunctions of following; meanwhile, β-ketoreduction and *N*-demetilation are regarded as dominative [13]. As a result of β-ketoreduction (the ketone group becomes reduced to hydroxyl group), dihydro-4-BMC is obtained. Within *N*-demetilation, a secondary amine is transformed into the primary amine, forming *N*-desmetylo-4-BMC. The combination of both reactions leads to *N*-demetylo-4-BMC formation.

However, the toxicological data concerning fatal 4-BMC intoxications remain unavailable, even though the forensic relevance increases, just like its availability on the illicit market.

Despite the escalating importance of synthetic cathinones abuse, available literature still lacks granular toxological data obtained from postmortem toxological investigations following 4-BMC. According to what we know, there has not been a reported lethal intoxication with this compound yet. Such paucity of data significantly restrains the results’ interpretation in both forensic and clinical toxicology. The range of blood 4-BMC concentrations that might involve the risk of fatal intoxication has not been determined yet, as well as the distribution of the substance within the tissues or its potential interactions with other xenobiotics. In this particular context concomitant use of 4-BMC with alcohol may be substantial. Ethanol acts as a competitive alcohol dehydrogenase (ADH) inhibitor. That is why such simultaneous exposure might have an impact on both a longer maintenance of the substance in the organism and its higher blood concentration level. Yet, this hypothesis still requires some further verification and experimental research.

Furthermore, insufficient detection of SCs in routine screening tests remains a major restraint. It may result from the limited availability of validated analytical methods and reference materials to primary compounds and their metabolites. Consequently, simultaneous identification of these substances remains constricted in both samples collected for judicial proceedings and those analyzed within the clinical diagnostics.

Therefore, the aim of the present study was to develop and validate an ultra-high-performance liquid chromatography–triple quadrupole tandem mass spectrometry (UHPLC-QqQ-MS/MS) method for the quantitative determination of 4-BMC in postmortem specimens and to apply this method to investigate the first reported fatal intoxication involving brephedrone. In addition to presenting the analytical findings, the study discusses the toxicological importance of the detected concentrations within the context of current knowledge regarding synthetic cathinones and the recent re-emergence of 4-BMC on the European drug market.

## 2. Materials and Methods

### 2.1. Case Report and Pathological Findings

In mid-2025, police officers were notified by a hotel employee that a 20-year-old male had been found unresponsive on the floor of a rented hotel room. Upon entering the room, the decedent was observed lying on the floor between two beds without signs of life. Emergency medical personnel arrived at the scene, and a physician performed an initial external examination. Early postmortem changes were present and fully developed, suggesting that death had occurred approximately 12–18 h before the commencement of the scene investigation.

No obvious signs of fatal trauma were observed during the preliminary examination. However, due to the young age of the deceased and the initially unknown cause of death, the possibility of third-party involvement could not be excluded at that stage of the investigation. The room did not show evidence of forced entry, significant disturbance, or indications of a struggle.

During the scene investigation, police officers secured several items of potential toxicological relevance. An electronic scale was found on a kitchen countertop together with a plastic bag containing compacted green-brown plant material. An additional plastic bag containing approximately 10 g of green herbal material was discovered among the decedent’s personal belongings, concealed inside a package of paper tissues. A rapid immunochemical screening test performed on the seized material yielded a positive result for cannabinoids (THC).

Police officers subsequently interviewed the decedent’s mother. According to her statement, she had spoken with her son during the afternoon of the day preceding his death. During the conversation, she did not notice any signs suggestive of intoxication, abnormal behaviour, or health complaints. She reported that the decedent had no known chronic medical conditions, was not receiving regular pharmacological treatment, and had no history of psychiatric illness. Furthermore, she stated that he was not known to abuse alcohol or illicit drugs. The man regularly participated in boxing training, maintained an active lifestyle, and paid attention to proper nutrition.

A forensic autopsy was performed the following day. Biological specimens collected for toxicological examination included postmortem whole blood and urine.

The urine and peripheral blood from the femoral vein were obtained directly during the autopsy performed in 2025. The biological material was put to test tubes containing sodium fluoride (NaF) as an anticoagulant. Right after the samples collection, all of them were transferred to controlled-temperature fridge and here is where they had been kept at a temperature of +4 °C until their analysis occurred. To mitigate the risk of analyzed compounds’ degradation, soon after the postmortem, the toxological investigation was conducted. Due to not a long time of pre-analysis sample storage, the impact of eventual degradation on assessed 4-BMC concentration in blood and urine can be presumed as negligible.

External examination revealed only a hemorrhagic infiltration within the subcutaneous tissue of the head. No injuries indicating severe blunt-force trauma, penetrating trauma, or other forms of external violence were identified.

Internal examination demonstrated cerebral edema and pulmonary edema. Petechial hemorrhages were observed beneath the pleura and epicardium, representing nonspecific findings commonly associated with acute cardiorespiratory failure. No significant pathological changes of the cardiovascular, respiratory, gastrointestinal, or central nervous systems capable of independently explaining death were identified. The hemorrhagic infiltration of the scalp was considered compatible with an uncontrolled fall and impact against a hard surface and was not regarded as contributory to the mechanism of death.

Overall, neither the external nor internal examination revealed a definitive anatomical cause of death. In particular, no evidence of significant natural disease, lethal traumatic injury, or pathological condition capable of explaining the fatal outcome was identified. Consequently, comprehensive toxicological investigations were considered necessary to determine whether xenobiotics contributed to or caused the death.

The toxicological examination included analyses for ethanol, therapeutic drugs, drugs of abuse, and a broad panel of new psychoactive substances. Screening and confirmatory analyses were performed using validated chromatographic and mass spectrometric methods routinely employed in forensic toxicology laboratories. The authors do not possess any knowledge of whether the histopathological research has been commissioned.

### 2.2. Chemicals

Water, acetonitrile, methanol, ethyl acetate (chemsolve^®^ LC–MS) were purchased from WITKO (Łódź, Poland); formic acid and ammonium formate were purchased from Chem-Lab NV (Zedelgem, Belgium); ammonium carbonate was purchased from Fluka (Buchs, Switzerland); mephedrone-*d*_3_ was purchased from Cerilliant (Round Rock, TX, USA); 4-bromomethcathinone was purchased from Chiron (Trondheim, Norway).

### 2.3. Biological Materials and Samples Procedure

Post-mortem whole blood and urine samples were sent to the Institute of Toxicology Research (Wrocław, Poland) for toxicological analysis to detect ethyl alcohol, drugs, pharmaceuticals, and new psychoactive substances (NPSs), in accordance with the current Polish legislation.

Analyses for ethyl alcohol were performed using a Shimadzu GC-2010 Plus AF IVD gas chromatograph (Shimadzu, Kyoto, Japan) equipped with two flame ionization detectors (FIDs). A Shimadzu HS-20 headspace autosampler (Shimadzu, Kyoto, Japan) was used for sample incubation and automated transfer of the headspace vapour to the GC system. The headspace effluent was equally divided (1:1) using a SilFlow microfluidic splitter (Trajan, Ringwood, Victoria, Australia) and introduced simultaneously onto two capillary columns: Zebron ZB-BAC1 (30 m × 0.32 mm i.d., 1.8 μm film thickness) and Zebron ZB-BAC2 (30 m × 0.32 mm i.d., 1.2 μm film thickness) (Phenomenex, Torrance, CA, USA), each connected to a separate FID. Helium (purity 99.9999%; from Messer) was used as the carrier gas. Headspace conditions were as follows: oven temperature, 65 °C; sample loop and transfer line temperatures, 150 °C; gas pressure, 60 kPa; and injection time, 1 min. Chromatographic separation was performed under isothermal conditions at 40 °C with a carrier gas flow rate of 2.57 mL/min. The FIDs were maintained at 240 °C, with helium, hydrogen, and air flow rates of 30, 40, and 400 mL/min, respectively. The total analysis time was 9 min.

For the determination of ethyl alcohol, 0.1 mL of each biological fluid (blood and urine) was placed into a 10 mL headspace vial (Alwsci Technologies, Shaoxing, China) following 0.5 mL of an internal standard solution (aqueous tert-butanol in concentration of 0.5 mg/mL). The vials were sealed with headspace caps (aluminum crimp cap with PTFE/Silicone septum; Alwsci Technologies, Shaoxing, China) and vortexed for 5 s.

Toxicological screening for drugs, pharmaceuticals, and NPSs was conducted using ultra-high-performance liquid chromatography coupled with triple quadrupole tandem mass spectrometry (UHPLC-QqQ-MS/MS).

Biological samples underwent a routine sample preparation procedure based on an established liquid–liquid extraction (LLE) method, involving 0.2 mL of the biological matrix, 0.02 mL of appropriate internal standards (ISTDs; e.g., amphetamine-*d*_11_, diazepam-*d*_5_, and in confirmation method, mephedrone-*d*_3_), 0.2 mL of 0.5 M ammonium carbonate (pH 9), and 2.0 mL of ethyl acetate. Samples were vortexed for 10 min, then centrifuged at 2540× *g* at 4 °C for 10 min. The supernatant was transferred to a 2 mL Eppendorf tube and evaporated under a gentle stream of nitrogen at 40 °C. The dried extract was reconstituted in 0.05 mL of methanol and transferred into a glass insert of an autosampler vial. The injection volume was 2 µL.

Due to the 4-BMC concentration exceeding the upper limit of quantification (ULOQ), quantitative analysis required a 100-fold dilution of both matrices. During validation, the dilution effect was assessed, and the calculated error remained within the predefined acceptable limits.

The method was validated in accordance with the Scientific Working Group for Forensic Toxicology (SWGTOX) standard practices for method validation in forensic toxicology [14] and Matuszewski et al. [15].

Calibration points and quality control (QC) samples were prepared by diluting the appropriate working solution with blank blood. The final concentrations of the calibrators were: 1 (LLOQ), 2, 5, 10, 20, 50, 80, 100, and 200 (ULOQ) ng/mL. QC samples were prepared by spiking blank blood with working solutions to yield final concentrations of: 1, 10, and 200 ng/mL.

The LOD was established at the laboratory’s Decision Point Concentration (DPC), corresponding to the administratively defined reporting threshold for this analyte, although a lower analytical LOD could be achieved. Similarly, the LLOQ was set at the Decision Point Concentration, based on the laboratory’s administratively defined reporting threshold, despite the method being capable of quantifying lower concentrations.

Precision was expressed as the coefficient of variation (%CV). For each concentration, the mean response and standard deviation (SD) were calculated to determine the %CV. The acceptance criterion was ±20%. Intra-day (within-run) precision was evaluated separately for each concentration in each of the five analytical runs, whereas inter-day (between-run) precision was calculated for each concentration across all five runs performed on five consecutive days.

Accuracy (bias) was calculated according to the following equation: Accuracy (%) = [(Mean measured concentration − Nominal concentration)/Nominal concentration] × 100. The acceptance criterion was ±20%. Both intra-day and inter-day accuracy were determined at each of the three quality control concentrations using the same experimental design as that applied for precision.

Matrix effects were evaluated at concentrations of 1, 10, and 200 ng/mL (*n* = 5). They were determined by comparing the peak areas of the analyte and internal standard obtained from neat standard solutions (A) with those obtained from blank blood or urine samples spiked after extraction (B). Matrix effects were calculated according to the method described by Matuszewski et al. [15]: Matrix effect (%) = (B/A) × 100.

Extraction recovery (*n* = 5) was assessed at concentrations of 1, 10, and 200 ng/mL. Recovery was calculated by comparing the mean peak areas obtained from samples spiked with the analyte and internal standard before extraction with those obtained from blank matrix samples spiked after extraction, according to the procedure described by [15].

Process efficiency was calculated for each quality control sample as the product of the absolute matrix effect and extraction recovery, according to the approach described by [15].

Dilution integrity was evaluated by fortifying blank blood and urine with the analyte to obtain a concentration of 10 µg/mL. The samples were subsequently diluted 100-fold with the corresponding blank biological matrix (blood or urine) and processed according to the validated sample preparation procedure. Concentrations were determined using the calibration curve, corrected for the dilution factor, and compared with the expected concentrations. The percentage error was calculated to assess dilution integrity.

Validation criteria were fully met for 4-BMC (for details see Table 1).

### 2.4. Chromatographic and Spectrometric Conditions

The prepared samples were analyzed using previously developed and validated UHPLC-QqQ-MS/MS methods (UHPLC, Shimadzu Nexera LC-40D XS, Kyoto, Japan) coupled with triple-quadrupole tandem mass spectrometry (LCMS-8060, Shimadzu, Kyoto, Japan) operating in positive ionization and multiple reaction monitoring (MRM) mode, as described elsewhere [16,17,18].

Because bromomethcathinone positional isomers may produce similar precursor and product ions, chromatographic differentiation was necessary to confirm the specific isomer detected in the biological specimens. To verify the specific isomer of 4-BMC, an alternative chromatographic method was developed. The separation was performed on a Kinetex^®^ Biphenyl 100 Å, 2.6 μm, 2.1 × 50 mm column (Phenomenex, Torrance, CA, USA), with the column oven maintained at 40 °C. The mobile phase consisted of 10 mM ammonium formate in water with 0.1% formic acid (A) and 10 mM ammonium formate in methanol with 0.1% formic acid (B). Gradient elution was applied at a constant flow rate of 0.25 mL/min according to the following programme: 0 min, 12% B; 16.00 min, 18% B; 16.01 min, 95% B; 17.50 min, 95% B; 17.51 min, 5% B; 20.00 min, 12% B; with these conditions maintained for an additional 6.0 min (total run time: 26 min).

The MS operating parameters were as follows: nebulizing gas flow, 3 L/min; heating gas flow, 10 L/min; drying gas flow, 10 L/min; interface temperature, 300 °C; desolvation line (DL) temperature, 250 °C; and heat block temperature, 400 °C. MRM conditions are shown in Table 2.

## 3. Results

### 3.1. Methods

**Figure 1:**
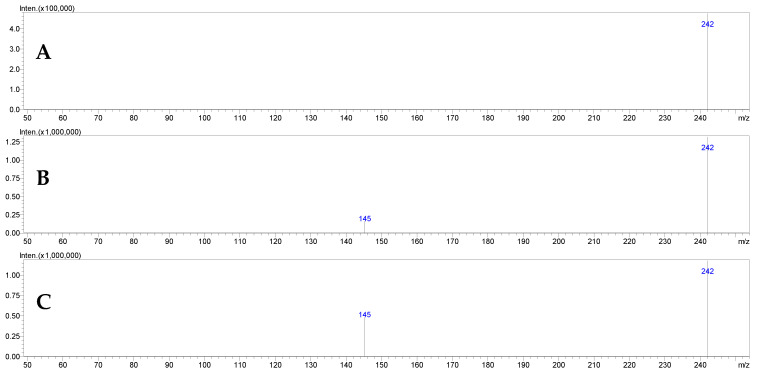

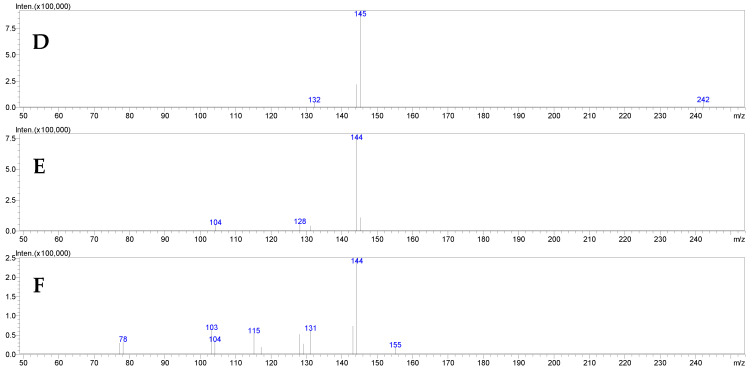
Mass spectra of 4-BMC in the studied blood sample (**A**) (100-fold dilution); collision energy (CE): 0 eV (**A**), 5 eV (**B**), 10 eV (**C**), 20 eV (**D**), 35 eV (**E**), and 45 eV (**F**).

One of the elements confirming the presence of 4-BMC in the biological materials was the comparison of the product ion scan spectrum obtained from the examined blood sample (diluted 1:100) with that of a QC sample (blank blood spiked with the analytical standard of 4-BMC at a concentration of 10 µg/mL, also diluted 1:100). Figure 1 presents the mass spectra for 4-BMC obtained from the analyzed blood sample.

**Figure 2:**
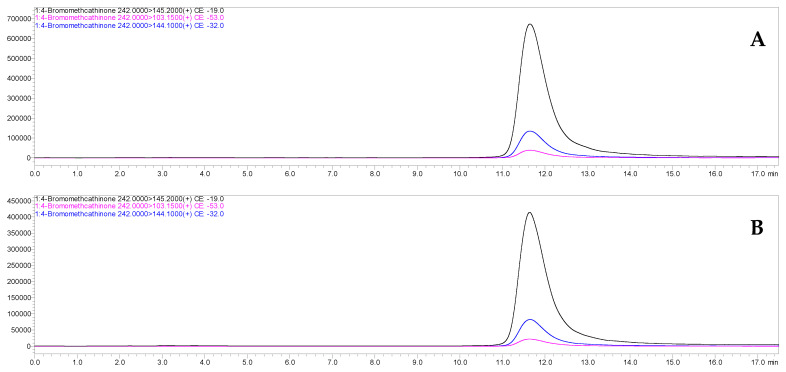
Comparison of MRM chromatograms of the substance in the studied blood sample (**A**) (100-fold dilution) and (**B**) the QC sample (10 µg/mL of 4-BMC in blank blood, diluted 100-fold), obtained using the developed method for isomer differentiation.

The developed method confirmed that the detected bromomethcathinone was 4-BMC. Figure 2 presents a comparison between the analyzed blood sample (100-fold dilution) and the QC sample, consisting of blank blood spiked with the analytical standard of 4-BMC at a concentration of 10 µg/mL (also diluted 100-fold).

### 3.2. Toxicological Results

In the described case, the concentration of 4-BMC was 16,601 ng/mL in blood and over 50,000 ng/mL in urine. In addition, 3-chloromethcathinone was determined in both matrices, reaching a concentration of 1.7 ng/mL in blood and 122.2 ng/mL in urine. Apart from the metabolites of BMC and CMC, no other xenobiotics or new psychoactive substances (NPSs) were detected. The results of the toxicological analyses are summarized in Table 3.

Furthermore, toxicological screening for ethyl alcohol in the blood yielded negative results.

## 4. Discussion

Synthetic cathinones remain among the most dynamically changing groups of new psychoactive substances encountered in forensic and clinical toxicology. Their continuous emergence, structural diversification, and changing patterns of availability substantially complicate toxicological screening and interpretation [1,2,5,20,21,22]. Although many synthetic cathinones disappear from the illicit market shortly after their first identification, some compounds may re-emerge after several years, sometimes in large quantities and with renewed toxicological relevance. By the end of 2021, the European Monitoring Centre for Drugs and Drug Addiction (EMCDDA; currently the European Union Drugs Agency, EUDA) had been monitoring approximately 880 NPSs. Only in 2020, 65% of reported NPS seizures constituted synthetic cathinones [3]. The scale of the European cathinone market has increased dramatically. During the past few years, the extensiveness of the European cathinone market has increased dramatically. According to EUDA data, at least 37 tonnes of synthetic cathinones were seized in Europe in 2023, compared with 26.5 tonnes in 2022, 8.5 tonnes in 2021, and only 0.7 tonnes in 2020 [4]. According to recent reports, synthetic cathinones still place among the most frequently detected NPSs within the European illicit drug market. Moreover, these compounds keep representing a major challenge for forensic laboratories, healthcare providers and law-enforcement agencies [4].

The present case illustrates this phenomenon particularly well, as 4-bromomethcathinone has been known in Europe since 2011 but has only recently attracted renewed attention due to its increased availability and seizures [9].

4-BMC has recently been subject to increased monitoring and risk assessment at the European level, together with other synthetic cathinones such as 2-MMC and NEP [4,9]. These data indicate that brephedrone should no longer be regarded only as an older or marginally relevant NPS, but rather as a re-emerging synthetic cathinone with potential clinical and forensic importance. In this context, the present fatal case is of particular significance because it provides the first postmortem concentration data associated with death following 4-BMC exposure.

According to demographic studies, young adult males between the ages of 18 and 35 tend to be the predominant target group for synthetic cathinones [23,24,25,26,27]. What accounts for the popularity of synthetic cathinones is their easy availability, relatively low cost and simple curiosity. Above and beyond, there is a common, but certainly erroneous conviction that NPSs are safer or legally acceptable alternatives to traditional illicit drugs [28,29,30]. The reasons that users frequently report consuming synthetic cathinones for are to enhance social interactions, increase sexual performance, improve mood or achieve stimulant effects comparable to those produced by cocaine or amphetamine derivatives [31]. Effects just like euphoria, increased sociability, elevated self-confidence, enhanced libido, increased motivation, decreased appetite, insomnia and psychomotor stimulation are those desired and seeked by the users [6,32,33]. However, depending on the chemical structure, dose, route of administration and concomitant use of other psychoactive substances, the pharmacological effects may vary strikingly.

Information concerning human exposure to 4-BMC remains limited. Most available data are derived from internet-based self-reports, seizure data, and a small number of non-fatal intoxication reports [7,8,9,16,19,20,31,34,35,36,37,38,39,40,41,42,43,44,45,46,47]. Users have described oral and intranasal administration as the most common routes of intake, although intravenous use has also been reported for synthetic cathinones [31,48,49]. Reported doses of 4-BMC range from approximately 50 to 400 mg, with lower doses associated mainly with mood enhancement, increased sociability, and stimulant effects, whereas higher doses have been linked to anxiety, insomnia, cardiovascular symptoms, muscle tension, headaches, and psychotic manifestations [31]. Such effects are consistent with the known pharmacology of synthetic cathinones, which primarily act through monoaminergic mechanisms involving dopamine, norepinephrine, and serotonin transporters [5,6].

The clinical picture of synthetic cathinone intoxication is often nonspecific and may include sympathomimetic, hallucinogenic, and serotonergic features [20,34]. Severe intoxications may progress to hyperthermia, seizures, rhabdomyolysis, acute kidney injury, cardiovascular collapse, cerebral edema, pulmonary edema, multiorgan failure, and death [19,20,28,34,35,36,37]. Fatal outcomes associated with synthetic cathinones may result directly from acute toxicity, suicide related to severe psychiatric disturbances, or accidents occurring under the influence of these substances [20,38,39,40]. In postmortem cases, autopsy findings are frequently nonspecific and may include pulmonary edema, cerebral edema, visceral congestion, and petechial hemorrhages, which require careful toxicological correlation. In the present case, the autopsy revealed cerebral and pulmonary edema and nonspecific signs of acute cardiorespiratory failure, while no significant traumatic injuries or natural disease processes capable of explaining death were identified. These findings are consistent with a fatal acute intoxication mechanism rather than a traumatic or natural cause of death.

Several fatal and non-fatal intoxications involving chloromethcathinones (CMCs) have previously been described [16,41,42,43,44,45,46,50]. Many of these cases involved polydrug exposure, including combinations with *N*-ethylpentylone [41], U-47700 [16], THC, MDMA, amphetamine, diazepam [50], and ethanol [43]. In contrast, fatal intoxications involving 4-BMC have not been reported previously.

The interpretation of the toxicological results in the present case is strengthened by the absence of major confounding factors. Toxicological screening did not reveal ethanol in blood, and no other drugs, medications, or new psychoactive substances were detected, apart from 4-BMC, trace 3-CMC, and their metabolites. The concentration of 4-BMC was very high, reaching 16,601 ng/mL in blood and exceeding 50,000 ng/mL in urine. In contrast, 3-CMC was present at only 1.7 ng/mL in blood and 122.2 ng/mL in urine. Considering the very low blood concentration of 3-CMC, its contribution to death was most likely minor or negligible. Therefore, the fatal outcome should be interpreted primarily in relation to acute 4-BMC intoxication.

To date, only three acute intoxications involving suspected 4-BMC exposure have been reported by Sweden and the Netherlands between 2014 and 2025 [9]. One of these cases was classified as life-threatening and required intensive care treatment. Moreover, the literature data concerning analytical confirmation of 4-BMC in biological matrices remain extremely limited. According to currently available EUDA data, only fifteen detections have been documented, including ten from Hungary and five from Sweden, with the majority reported in 2024 [9].

Because no fatal cases involving 4-BMC have previously been published, interpretation of the blood concentration detected in this case must rely on comparison with structurally and pharmacologically related synthetic cathinones. Fatal intoxications involving chloromethcathinones and methylmethcathinones have been reported across a wide concentration range, reflecting differences in individual tolerance, route of administration, co-ingested substances, postmortem redistribution, degradation, and the interval between death and sample collection [16,41,42,43,44,45,50,51]. Adamowicz reported blood concentrations of synthetic cathinones in fatal and non-fatal cases and demonstrated the broad variability of concentrations encountered in forensic toxicology [44]. Tusiewicz et al. described a fatal case involving an exceptionally high 4-CMC concentration [45]. More recently, Kriikku and Ojanperä analyzed synthetic cathinone findings in postmortem toxicology, further confirming that interpretation of cathinone concentrations requires case-specific evaluation rather than simple comparison with fixed toxic or fatal ranges [51]. In this context, the 4-BMC blood concentration of 16,601 ng/mL observed in the present case should be considered consistent with fatal intoxication.

The simultaneous detection of 3-CMC deserves additional comment. Chloromethcathinone isomers, particularly 3-CMC and 4-CMC, are currently among the most relevant synthetic cathinones in European forensic casework [4,9,13,19]. Romańczuk et al. highlighted interpretative problems related to the presence of chloromethcathinone isomers in postmortem material and emphasized the value of dihydro-CMC as a biomarker of intake, especially when the parent compound is unstable or no longer detectable [19]. In the present case, however, 3-CMC was detected only at a trace concentration in blood. This finding may indicate previous or minor co-exposure, contamination of the consumed product, or intake of a preparation containing more than one cathinone. Nevertheless, the quantitative disproportion between 4-BMC and 3-CMC strongly supports 4-BMC as the principal toxicologically relevant compound.

From an analytical perspective, the present case also demonstrates the importance of reliable differentiation of positional isomers. Bromomethcathinone isomers may share the same nominal mass and produce similar product ions, making identification based solely on selected MRM transitions potentially insufficient. Therefore, chromatographic separation and comparison with certified reference material are essential for unambiguous identification. In the present study, an additional chromatographic method using a biphenyl stationary phase was applied to confirm the presence of 4-BMC and exclude potential misidentification with other bromomethcathinone isomers. This is particularly important in forensic toxicology, where incorrect isomer assignment may affect both toxicological interpretation and legal classification.

Analyte stability represents one of the most important limitations in the interpretation of synthetic cathinone concentrations. Previous studies investigating the stability of synthetic cathinones demonstrated that many compounds from this group exhibit limited stability in biological materials, and that their degradation depends on several factors, including matrix type, pH, storage temperature, storage time, preservative use, initial concentration, and chemical structure [52,53,54,55,56,57]. Although no studies concerning the stability of 4-BMC in biological matrices are currently available, it may be assumed that brephedrone behaves similarly to structurally related para-substituted cathinones such as 4-CMC, 4-MMC, and 4-FMC. Consequently, it is highly probable that the concentration of 4-BMC in the decedent’s body at the time of death was higher than the concentration determined in the analyzed postmortem specimens.

Aldubayyan et al. reviewed the stability of synthetic cathinones and their metabolites in clinical and forensic toxicology and emphasized that instability remains a major source of uncertainty in interpretation [57]. More recently, Aldubayyan et al. investigated the long-term stability of synthetic cathinones and dihydro-metabolites in human urine under different storage conditions [58]. This new work is important because it highlights not only the instability of parent cathinones, but also the potential usefulness of more stable metabolites as markers of intake [58]. Together with the findings of Romańczuk et al. concerning dihydro-CMC [19], these data support a metabolite-oriented approach in the interpretation of synthetic cathinone cases. For 4-BMC, further studies are needed to establish whether specific reduced or demethylated metabolites may be more stable than the parent compound and therefore more reliable for documenting exposure in delayed or degraded postmortem specimens.

The present findings should therefore be interpreted with caution but also with appropriate toxicological weight. The detected blood concentration of 4-BMC was extremely high, no alternative cause of death was identified, and no other toxicologically significant xenobiotics were present. Even if some degradation occurred before analysis, this would only strengthen the interpretation that the perimortem concentration of 4-BMC was at least as high as, and possibly higher than, the measured postmortem value.

The case also has broader public health implications. The re-emergence of 4-BMC after more than a decade demonstrates that substances previously considered marginal or historical may return to the illicit market and cause severe or fatal intoxications. This phenomenon complicates routine toxicological screening because laboratories may remove older NPSs from active methods when their prevalence decreases. The present case indicates that screening panels should be periodically updated not only for newly emerging substances but also for re-emerging compounds documented by early warning systems. Inclusion of 4-BMC, its positional isomers, and relevant metabolites in targeted LC-MS/MS or LC-HRMS methods may improve detection of intoxications and contribute to more accurate monitoring of drug market trends.

## 5. Conclusions

This study describes the first reported fatal intoxication involving 4-bromomethcathinone (4-BMC) and the first report providing postmortem concentration data for this substance. The case highlights the increasing forensic relevance of 4-BMC as a re-emerging synthetic cathinone within the European illicit drug market and emphasizes the importance of including both newly emerging and previously established NPSs in routine toxicological screening strategies. Given the recent increase in 4-BMC seizures and its growing availability, additional intoxications and fatalities involving this compound may be expected in the future.

Furthermore, the findings underline the importance of accurate analytical differentiation of structurally related synthetic cathinones and support the growing role of metabolite-oriented approaches in forensic toxicology. Further studies investigating the pharmacokinetics, metabolism, stability, and toxicological significance of 4-BMC are needed to improve the interpretation of clinical and postmortem cases. Continuous monitoring of new psychoactive substances remains essential for identifying changes in drug markets, supporting public health initiatives, and improving forensic interpretation of intoxication-related deaths.

## Figures and Tables

**Table 1:** Validation results.

4-BMC		Blood
*R^2^*		>0.9999
LOD		0.5 ng/mL
LLOQ		1.0 ng/mL
ULOQ		200.0 ng/mL
Intra-day precision [%] *	1 ng/mL	8.3
	10 ng/mL	3.5
	200 ng/mL	3.7
Intra-day accuracy [%] *	1 ng/mL	8.2
	10 ng/mL	1.1
	200 ng/mL	−3.0
Inter-day precision [%] *	1 ng/mL	7.1
	10 ng/mL	3.0
	200 ng/mL	5.0
Inter-day accuracy [%] *	1 ng/mL	8.4
	10 ng/mL	0.1
	200 ng/mL	−2.5
Recovery [%] *	1 ng/mL	100.2
	10 ng/mL	99.1
	200 ng/mL	100.5
Matrix effect [%] *	1 ng/mL	115.1
	10 ng/mL	109.2
	200 ng/mL	105.8
Process efficiency [%] *	1 ng/mL	114.9
	10 ng/mL	110.2
	200 ng/mL	105.3
Dilution effect **	Blood: 10 µg/mL, 100×	<5%
	Urine: 10 µg/mL, 100×	<5%

* *n* = 5; ** *n* = 2; LOD—limit of detection; LLOQ—lower limit of quantification; ULOQ—upper limit of quantification.

**Table 2:** MRM condition used in routine analyses of selected compounds. * with reference to the retention time in the additional method.

Compounds	Precursor Ion (*m*/*z*)	Product Ion (*m*/*z*)	Q1 Pre-Bias (V)	Collision Energy (V)	Q3 Pre-Bias (V)	Retention Time * (min)
4-Bromomethcathinone	242.00	145.20 103.15 144.10	10 17 11	19 53 32	14 17 14	4.19 11.72 *
3-Chloromethcathinone	198.00	145.20 180.20 144.10	11 11 10	20 13 29	14 18 23	3.94
Mephedrone-*d*_3_	181.00	148.20 163.20	27 23	23 16	24 16	3.73

**Table 3:** Toxicological results in the described case. * Mass spectra compared to studies [13,19].

Substance	Concentration in Blood [ng/mL]	Concentration in Urine [ng/mL]
4-BMC	16,601	>50,000
3-CMC	1.7	122.2
Metabolites of 4-BMC *	detected	detected
Metabolites of 3-CMC *	detected	detected
NPSs	not detected	not detected
Ethanol	not detected	0.11 [‰]

## Data Availability

The data that supports the findings in this study are available from the corresponding authors upon reasonable request.

## Supplementary Materials

The following supporting information can be downloaded at: https://www.mdpi.com/article/10.3390/metabo16070507/s1, Figure S1: Structures of 2-, 3-, and 4- isomers of: methcathinone, bromomethcathinone, chloromethcathinone and fluoromethcathinone.

## Abbreviations

The following abbreviations are used in this manuscript:

2-BMC	2-bromomethcathinone
3-BMC	3-bromomethcathinone
4-BMC	4-bromomethcathinone
3-CMC	3-chloromethcathinone
4-CMC	4-chloromethcathinone
4-FMC	4-fluoromethcathinone
2-MMC	2-methylmethcathinone
3-MMC	3-methylmethcathinone
4-MMC	4-methylmethcathinone
CE	collision energy
%CV	coefficient of variation
DAT	dopamine transporter
DPC	Decision Point Concentration
EMCDDA	European Monitoring Centre for Drugs and Drug Addiction
ESI	electrospray ionization
EUDA	European Union Drug Agency
FID	flame ionization detector
GC	gas chromatography
HS-GC-FID/FID	Headspace Gas Chromatography with Dual Flame Ionization Detection
ISTDs	internal standards
LC-HRMS	Liquid Chromatography-High Resolution Mass Spectrometry
LC-MS/MS	Liquid Chromatography-Tandem Mass Spectrometry
LLE	liquid–liquid extraction
LLOQ	lower limit of quantification
LOD	limit of detection
MDMA	3,4-methylenedioxymethamphetamine
MRM	multiple reaction monitoring
NET	norepinephrine transporter
NPSs	new psychoactive substances
QC	quality control
SCs	synthetic cathinones
SD	standard deviation
SERT	serotonin transporter
SWGTOX	the Scientific Working Group for Forensic Toxicology
THC	Δ-9-tetrahydrocannabinol
UHPLC-QqQ-MS/MS	Ultra-High Performance Liquid Chromatography coupled with Triple Quadrupole Mass Spectrometry
ULOQ	upper limit of quantification
