# Supplementary material for: 4-Bromomethcathinone (4-BMC) Revisited: The First Reported Fatal Case and Toxicological Insights
Source: Metabolites. 2026 Jul 21;16(7):507. doi: 10.3390/metabo16070507 (PMC13413749; doi:10.3390/metabo16070507)
Supplement: Supplementary file 1 [file metabolites-16-00507-s001.zip › metabolites-4396260-supplementary.pdf]

Supplementary Materials

4-Bromomethcathinone (4-BMC) Revisited: The First Reported Fatal Case and Toxicological Insights

Karolina Nowak[1*,], Paweł Szpot[2,], Marcin Zawadzki[3] and Agnieszka Chłopaś-Konowałek[4,5]

[1] Department of Pharmacology, Faculty of Medicine, University of Opole, 48 Oleska Street, 45052 Opole, Poland; karolina.nowak@uni.opole.pl
[2] Department of Forensic Medicine, Faculty of Medicine, Wroclaw Medical University, 4 J. Mikulicza-Radeckiego Street, 50345 Wroclaw, Poland; pawel.szpot@umw.edu.pl
[3] Department of Social Sciences and Infectious Diseases, Faculty of Medicine, Wroclaw University of Science and Technology, 27 Wybrzeze Wyspianskiego Street, 50370 Wroclaw, Poland; m.zawadzki@pwr.edu.pl
[4] Department of Forensic Medicine, Division of Molecular Techniques, Wroclaw Medical University, Sklodowskiej-Curie 52, 50369 Wroclaw, Poland; agnieszka.chlopas-konowalek@umw.edu.pl
[5] Institute of Toxicology Research, 55/61/306 Curie-Sklodowskiej Street, 50369 Wroclaw, Poland
* Correspondence: karolina.nowak@uni.opole.pl

Figure S1. Structures of 2-, 3-, and 4- isomers of: methcathinone, bromomethcathinone, chloromethcathinone and fluoromethcathinone.

methylmethcathinone isomers

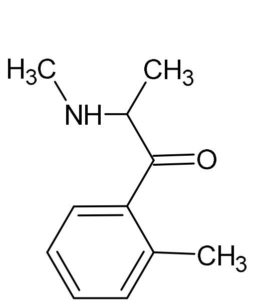

2-methylmethcathinone

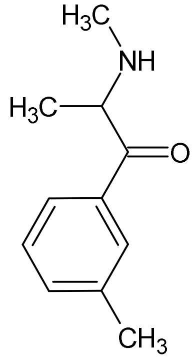

3-methylmethcathinone

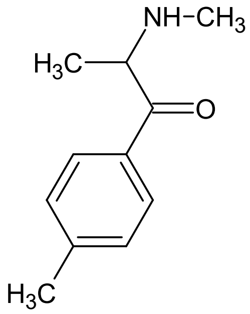

4-methylmethcathinone

bromomethcathinone isomers

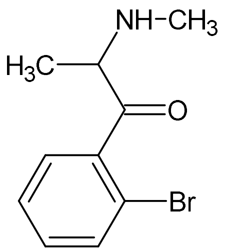

2-bromomethcathinone

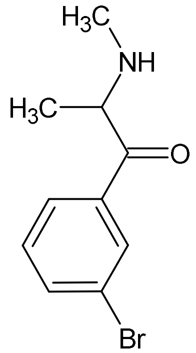

3-bromomethcathinone

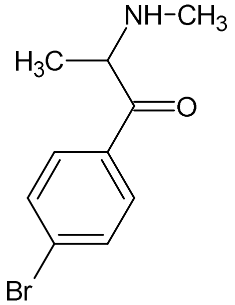

4-bromomethcathinone

chloromethcathinone isomers

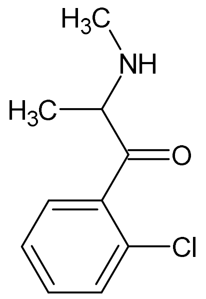

2-chloromethcathinone

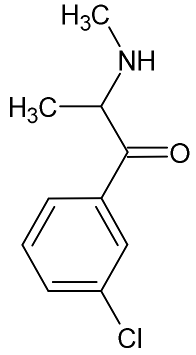

3-chloromethcathinone

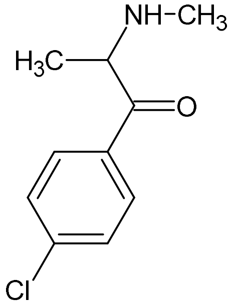

4-chloromethcathinone

fluoromethcathinone isomers

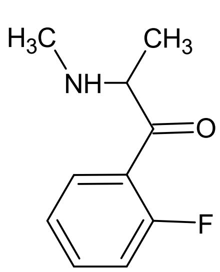

2-fluoromethcathinone

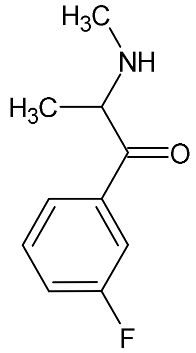

3-fluoromethcathinone

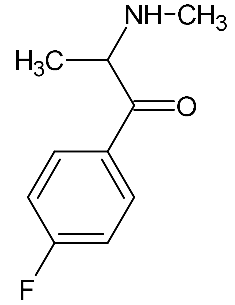

4-fluoromethcathinone
